# Supplementary material for: Three-dimensional skyrmionic cocoons in magnetic multilayers
Source: Nat Commun. 2022 Nov 11;13:6843. doi: 10.1038/s41467-022-34370-x (PMC9652402; doi:10.1038/s41467-022-34370-x)
Supplement: Supplementary file 1 — Supplementary Information [file 41467_2022_34370_MOESM1_ESM.pdf]

Three-dimensional skyrmionic cocoons in magnetic multilayers
Supplementary Information

Matthieu Grelier[1], Florian Godel[1], Aymeric Vecchiola[1], Sophie Collin[1], Karim Bouzehouane[1], Albert Fert[1], Vincent Cros[1], Nicolas Reyren[1]

[1] *Unité Mixte de Physique, CNRS, Thales, Université Paris-Saclay, 91767, Palaiseau, France.*

1 Multilayer architecture

The magnetic multilayers properties can be accurately tuned to answer specific requirements which is deeply interesting for the engineering of three-dimensional textures. In the main text, we have chosen to focus on the SG structure with a step thickness S of 0.1 nm but a thorough numerical study was done on other possible architectures. To investigate the magnetic textures that could be host in such structures, micromagnetic simulations were performed using *Mumax3* [1] for different thickness steps values, at various magnetic fields and X_1, while $N = 13$ layers was fixed. For each set of parameters, the magnetization was initially set out-of-plane (OOP) with some noise (50%) and then relaxed at each value of the increasing OOP magnetic field. Then, the average heights of all the textures present in the simulation space was computed as well as their average circularity C to differentiate the stripes states ($C < 0.9$) from the states presenting more circular and isolated objects ($C \geq 0.9$). The circularity is computed as $4 \times A \times \pi / P^2$ with A the area of the object and P its perimeter. In Fig. S1a,b, three representative cases are displayed: $S = 0$ nm, which corresponds to the uniform case, $S = 0.1$ and 0.2 nm, in which the average heights for different thicknesses and magnetic fields is presented. Those graphs evidence the impact of the thickness gradient: the larger S is, the more confined the textures can be at equivalent field and thickness. Indeed, with $S = 0.2$ nm, with fields stronger than 200 mT and an average thickness below 2 nm, some textures that are only present in half the overall thickness (less than 7 layers) can be stabilized whereas for the uniform case, an average height below 10 layers occurs infrequently. Similar conclusions can be drawn on the intermediate case, $S = 0.1$ nm, for which at high field we can reach more confined textures. To support this claim, vertical cut of the magnetization of an axisymmetric selected object are displayed below their respective graphs, for a magnetic field of 250 mT and an average thickness close to 2 nm. We observe that for $S = 0$ nm, it corresponds to a columnar skyrmion (Fig. S1a) while for the other two it disappears from the outer layers and acquire a characteristic ellipsoid shape. Moreover, the range of stability in thickness seems to be broaden by the presence of the gradients as we are able to stabilize textures with lower thicknesses than the uniform case. Finally, by introducing a variable thickness into the structure, it is possible to obtain states with isolated textures at lower field and thickness rather than stripes or worms as shown by the circularity. Thus, overall, the use of gradient facilitates the stabilization of isolated objects which can be strongly confined under the appropriate conditions.

We also consider what we call Reversed Single Gradient (RSG) with the inverse thickness evolution, i.e $S < 0$. For $S = -0.1$ nm, the impact of the gradient seem minimal: the confinement of the textures appears quite close to the uniform case. The magnetization cut shows also an object with a higher diameter

at equivalent field and average thickness. A stronger effect is noticeable when using a larger step but it is restricted to a small range of thicknesses and the majority of the relaxed magnetic states corresponds to stripe states and not isolated objects, thus limiting the interest of this other possible structure. Note that we observe a magnetic object split in half in the YZ cut, with the scission happening in the layer with the stronger OOP anisotropy (of thickness 1.2 nm in that case). It shows the importance of strong PMA layers in the manipulation of the 3D magnetization distribution. However, the object is less confined than with $S > 0$ and the bottom and top layers display a complex behavior due to the strong in-plane effective anisotropy and the dipolar field from the structure. Thus, based on those observations and the full numerical study, in this work we have chosen to focus on the intermediate case of SG: all results presented relate to $S = 0.1$ nm.

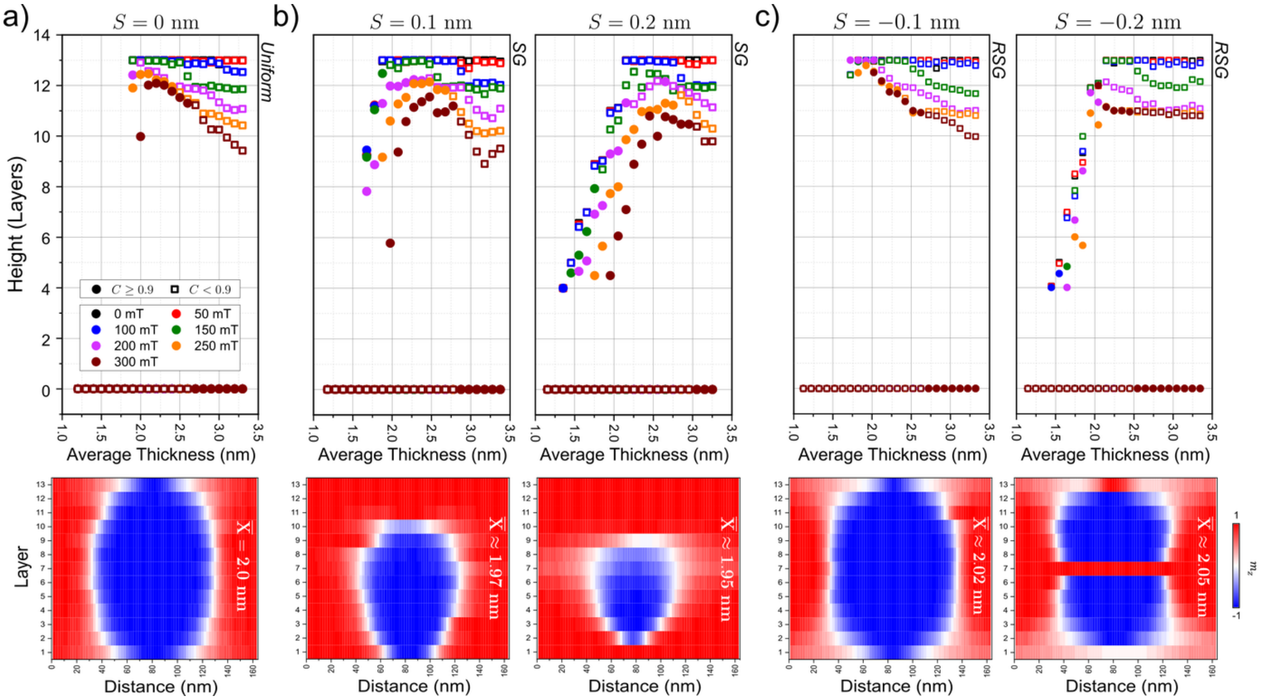

Figure S1: Average height of the magnetic textures obtained in micromagnetic simulations for the a) uniform, b) SG and c) RSG structures with various S parameters at different magnetic fields and thicknesses with $N = 13$ layers. Depending on the circularity of the textures, the marker is either an open square for $C < 0.9$ or a circle otherwise. A zero height corresponds to a uniform magnetization. Below each graph is an associated vertical cut of the magnetization of an axisymmetric object at an external field of 250 mT and an average thickness close to 2 nm.

2 Magnetic hysteresis

Field evolution The magnetic hysteresis of our samples have been measured using an alternating gradient field magnetometer (AGFM) and displayed in Fig. S2a. As the reorientation transition thickness from out-of-plane to in-plane anisotropy is 1.7 nm in our Pt|Co|Al trilayers, the multilayers under consideration would display an in-plane effective anisotropy in a uniform state, at the exception of the PMA layers. This is confirmed by the AGFM measurements. For the SG, the easy axis lies in-plane while for the DG the situation is more complex due to the presence of the PMA layers which keep an out-of-plane magnetization

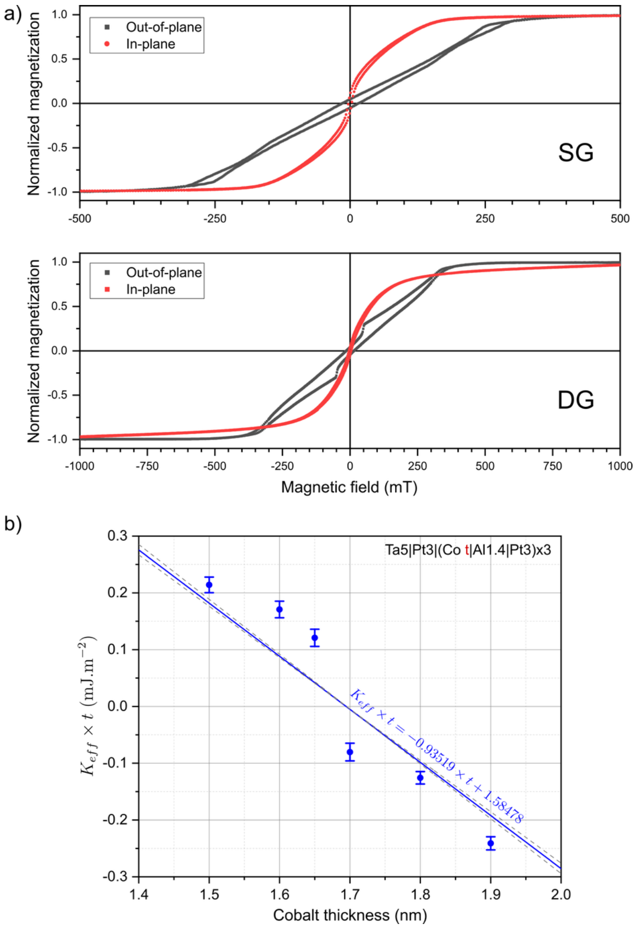

Figure S2: Magnetic characterization performed with alternating gradient force magnetometer (AGFM) measurements. a) Magnetic hysteresis for the SG and DG of the main text. b) Evolution of the effective anisotropy with the Cobalt thickness t in Ta5|Pt3|(Co t|Al1.4|Pt3)x3 multilayers. Error bars are uncertainties stemming from the estimation of the saturation field only. The blue line corresponds to a linear fit imposing the saturation magnetization measured with the SQUID: $M_S = 1.22$ MA/m while the gray dotted line are computed by using the lower and higher limit of the confidence interval of M_S.

up to higher fields. Near 70 mT with an out-of-plane (OOP) field coming from higher fields, the PMA layers partially switch and form some magnetic domains which yields a non-uniform state at remanence.

Effective anisotropy To estimate the surfacial uniaxial anisotropy $K_{u,s}$ of our multilayers, simpler structures have been studied made of three repetitions of (Co t|Al1.4|Pt3) where t corresponds to the Cobalt thickness. From AGFM measurement, the effective anisotropy K_{eff} was extracted which relates to $K_{u,s}$ with:

$$K_{\text{eff}}\ t = K_{u,s} - \frac{\mu_0\ M_S^2}{2}\ t \tag{1}$$

Using the M_S measured by SQUID (1.22 ± 0.02 MA/m), a linear fit yields $K_{u,s} = 1.58 \pm 0.08$ mJ/m^2 (see Fig. S2b) and a spin reorientation thickness of 1.7 nm ($K_{\text{eff}} = 0$ mJ/m^2).

3 Supplementary MFM images

SG samples In order to optimize the SG structures, various growths have been performed while changing the different parameters (S, N, or X_1). To display the impact of those parameters, we consider the magnetic states obtained at zero field as ideally we want to stabilize textures without any external magnetic field. Here, the samples magnetization have been saturated with an out-of-plane field of 1.8 T before being measured with MFM. By considering the variations of the thickness of the bottom layer X_1 between 1.6 nm and 2.1 nm with the other parameters fixed (Fig. S3a), if X_1 is too thick we mostly observe elongated objects and stripes which is coherent with the numerical study of those structures. Optimally, the thicknesses for which the textures have the highest circularity and density are 1.7 and 1.8 nm. Moreover, if we increase the thickness steps S while conserving the minimum thickness $X_1 = 1.8$ nm and the maximum one $X^{\max} = 2.4$ nm (Fig. S3b), similar observations can be made as the textures expands more and more when increasing S. The optimal value appears to be 0.1 or 0.15 nm when comparing samples with fixed X_1 and $X^{\max}$. Based on that experimental study and the numerical work, we focused on those SG to implement them in the DG structures.

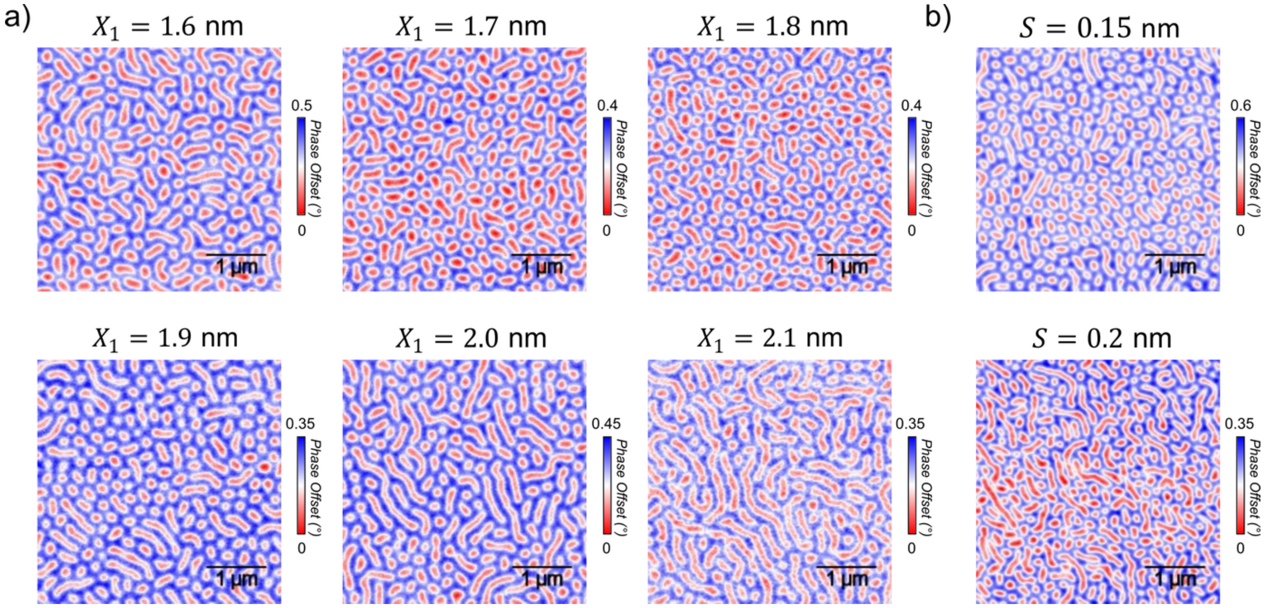

Figure S3: MFM phase maps of Single Gradient samples at zero field after an out-of-plane saturation (1.8 T). a) Variation of X_1 with the other parameters are fixed: $S = 0.1$ nm and $N = 13$ layer. b) Variations of S with $X_1 = 1.8$ nm and $X^{\max} = 2.4$ nm fixed.

DG samples The DG properties are quite sensitive to the number of layers with strong PMA M present in the structures. In Fig. S4a, we present MFM images of three different samples with respectively $M = 15, 10, 5$ repetitions with other parameters fixed. First, they were demagnetized with a field tilted 60 degrees away from the sample normal and then the field was increased to reduce the number of objects and finally it was decreased back down to observe the nucleation process. For $M = 15$ and $M = 10$, after the field reduction, cocoons have appeared among the columnar textures, although with different densities, contrarily to the $M = 5$ cases which shows that for reduced value of M, mostly columnar textures are forming and stable. Thus, it is possible to tune the relative density of cocoons and columnar skyrmions by adapting the

number of strong PMA layers.

In Fig. S4b, the same zone was scanned at different lift heights on a DG to probe the sensitivity of the MFM with respect to the bottom layers. We find that, for a lift height ranging from 5 to 80 nm, which corresponds roughly to the gradient thickness, the intensity of the signal for all the cocoon is typically divided by a factor 4 and their apparent size increases significantly. Thus, the signal of the cocoons is almost negligible and all have the same behavior. This suggests that the MFM is only picking up the signal of the top layers as expected from the large thickness of our sample and the rapid decrease of the stray field.

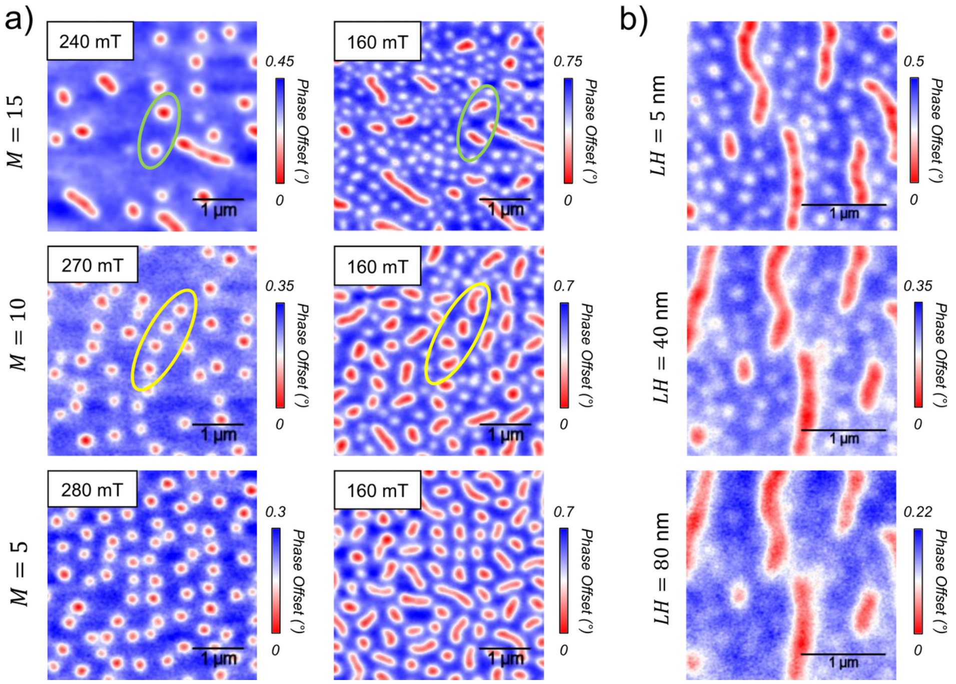

Figure S4: MFM phase maps of Double Gradients. a) Images of samples with parameters $X_1 = 1.7$ nm, $S = 0.1$ nm, $N = 13$ layers, $Y = 0.9$ nm and a various number of PMA layers M after a demagnetization with a field at 60 degrees away from the normal and following a similar magnetic history. The green ellipses encircles the same textures at different field. b) Images of the DG of the main text with parameters $X_1 = 2.0$ nm, $S = 0.1$ nm, $N = 13$ layers, $Y = 1.0$ nm and $M = 15$ repetitions at different lift heights at 265 mT.

4 Electronic transport

Simulation states As shown on Fig. 4 of the main text, the micromagnetic simulations are in agreement with the electronic transport measurements regarding the evolution of the longitudinal and transverse resistances. In Fig. S5, representative relaxed states from those simulations are displayed at different fields and angles, yielding complex magnetic textures for θ superior to 0°. For $\theta = 0°$, the evolution of the relaxed states correspond to the description of the main text. For $\theta = 30$, 60°, the field evolution are similar: at high field, stripes are located in the gradient parts of the DG until nucleation in the middle layers at 75 mT which yields elongated cocoons inside the 3D worms. For $\theta = 90°$, the initialization is different as it starts with an in-plane initialization which gives parallel stripes in the middle layers whereas the gradient parts are saturated at high positive field. When decreasing the field, the stripes consequently expend in the gradients

until they reach the surface at 0 mT. The inverse trend is shown when going down to negative field. In conjunction with the AFGM measurements, it is harder to saturate the strong PMA layers with an in-plane field so stripes are still present even at -500 mT.

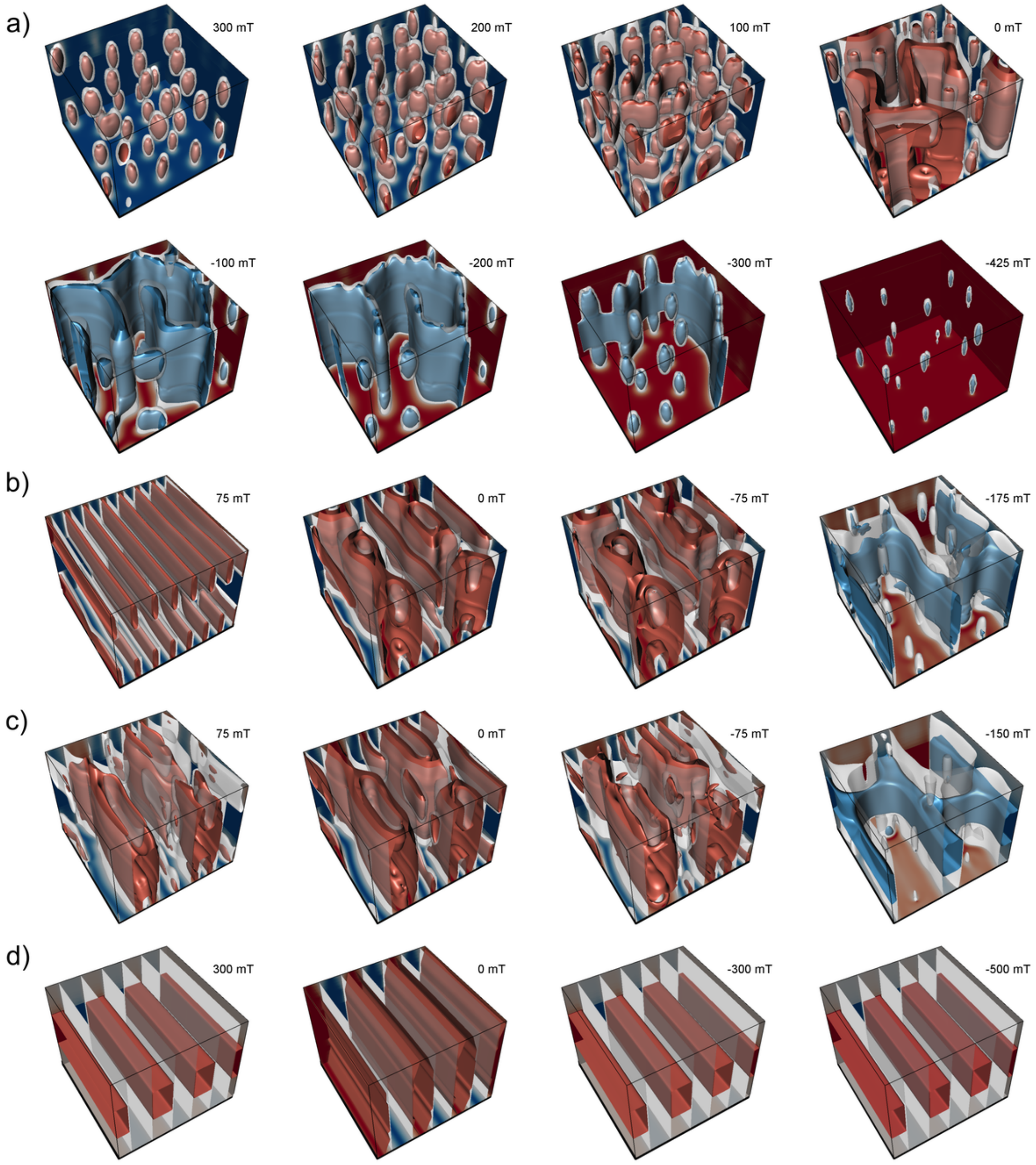

Figure S5: Three dimensional representation of the simulations for the transport measurements at different angles for the fields. With θ being the angle between the z-axis and the y-axis, the field is at a) $\theta = 0°$ (OOP), b) $\theta = 30°$, c) $\theta = 60°$, d) $\theta = 90°$. The isosurfaces corresponds to $m_z = -0.8$ in red, $m_z = 0$ in white and $m_z = 0.8$ in blue. Each simulation area corresponds to $1.024 \times 1.024\ \mu\text{m}^2$

Additional curves In Fig. S6a,b, the transverse and longitudinal resistances are displayed for the SG structure of the main text as a function of the external magnetic field with the corresponding micromagnetic simulations. An excellent correlation is again observed with the latter. A similar reasoning than for the DG can be held to understand the measured trends. For instance, for $\theta = 0°$, R_{xx} decreases when the cocoons nucleate and their expansion further lower it until it reaches its minimum at zero field. In Fig. S7c, the curves of R_{xy} for $\theta > 0°$ are displayed for the DG, showing the same characteristic jump for the strong PMA layers transition. Finally, in Fig. S7d, the angular scans are shown for both structures at field high enough to saturate the sample (650 mT for the SG and 3T for the DG). Their amplitude in the different planes allow to retrieve the experimental parameters R_{AMR} and R_{SMR}.

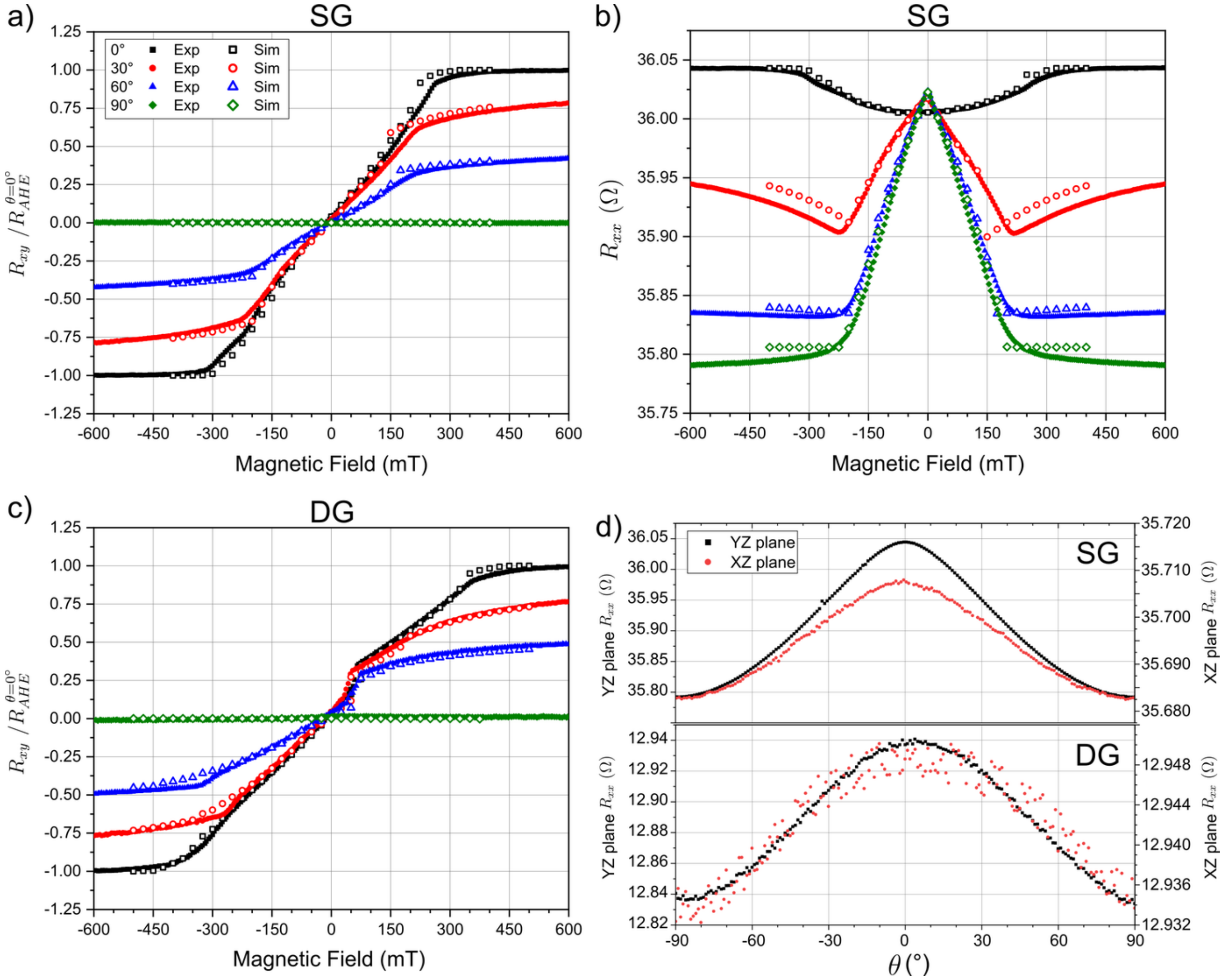

Figure S6: Electronic transport measurements on a SG structure ($X_1 = 1.7$ nm, $S = 0.1$ nm, $N = 13$ layers) on a 30×150 μm^2 Hall bars associated with the corresponding micromagnetic simulations showing a) R_{xy} (H) and b) R_{xx} (H). c) Complementary R_{xy} (H) measurements for the DG structure. The field is swept from positive to negative. d) Angular scans for both field planes for the SG at 650 mT and the DG at 3T.

Sensitivity to 3D evolution Due to the structure of the cocoons predicted by the micromagnetic simulations, it is important to estimate the sensitivity of the electronic transport measurements to the evolution

of the textures over the thickness. To that end, we consider the longitudinal resistance of a SG in presence of an out-of-plane decreasing magnetic field (Fig. S7a) and compare it to different states extracted from the micromagnetic simulations. We focus on the state at 200 mT because it displays an important evolution over the thickness and modify it to investigate four different states: the unchanged one (A) and some in which layers have been set uniformly up (B,C,D). This modification is performed over the whole simulations space and we show how it impacts a given texture with the magnetization cuts. Between the most different states A and D, a difference of 3 mΩ is obtained which is superior to the characteristic noise of our measurements. Between the closest states A and B, this difference drops to 0.6 mΩ which would be harder to resolve experimentally. Therefore, the transport measurement shows some sensitivity to the 3D profile but might lack precision depending on its complexity.

Similarly, in the DG, erasing the top cocoons leads to a significant difference in the calculated resistances, as shown in Fig. S7b. Therefore, we conclude from the match of the simulations with experiments (black points) that the cocoons must be present in both SG.

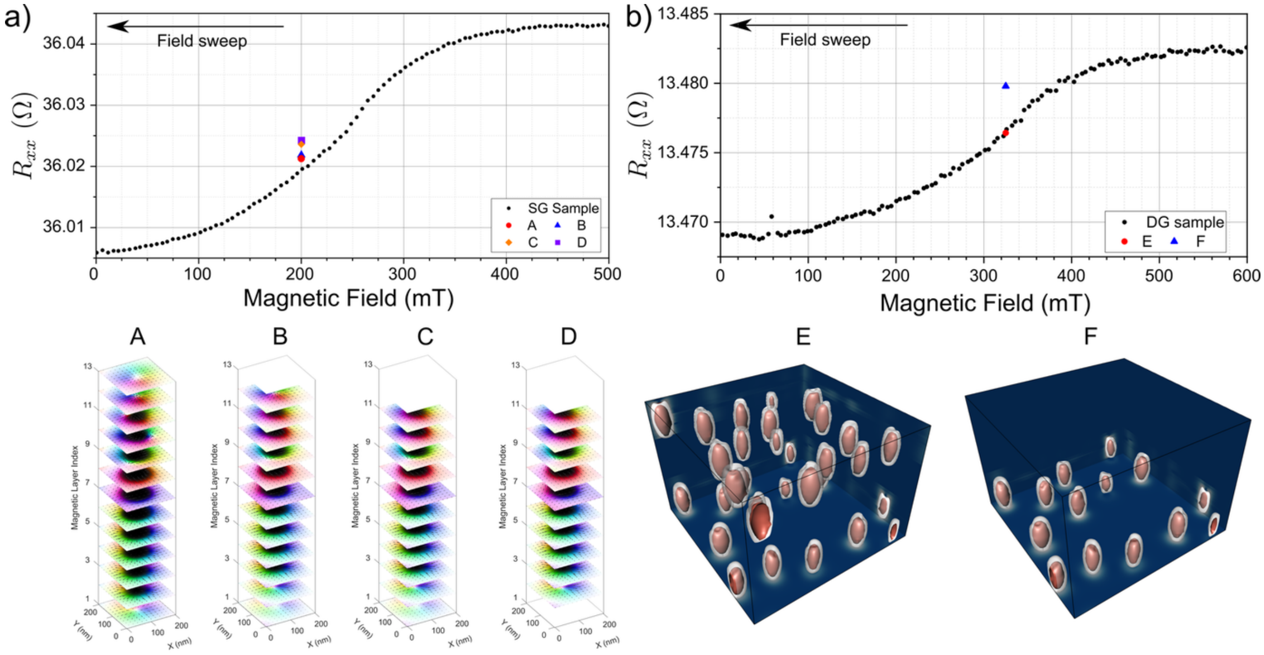

Figure S7: Sensitivity of the transport measurements. a) Longitudinal resistance R_{xx} measured on a SG (X_1 = 1.7 nm, S = 0.1 nm, N = 13 layers). The different points at 200 mT correspond to simulations whose states have been modified to test the sensitivity of the measurement regarding the textures' vertical profile.Those modifications are illustrated on a single texture with the various cuts of the magnetization displayed at the bottom. A state: unmodified. B (resp. C) state: top (resp. two) layer uniformly pointing up. D state: top two layers and bottom one uniformly pointing up. b) R_{xx} measured on a DG (X_1 = 2.0 nm, S = 0.1 nm, N = 13 layers and M = 15 repetitions) with numerical comparison at 325 mT for two states. E: unmodified. F: only the skyrmionic cocoons present in the bottom gradient.

5 Topology and field behavior of skyrmionic cocoons

Size evolution Skyrmionic cocoons display an important modulation over the thickness depending on the external magnetic field. In Fig. S8, we show map of the radius r_x along the x direction as a function of the field and the magnetic layer for two different textures identified on the magnetization cut on the right. Their evolution is quite similar even though their initial size differs: until 100 mT they remain fairly homogeneous over the thickness but increasing the field even more cause them to shrink significantly in the outer layers until they disappear from them. For instance, at 275 mT for both textures, they are not present in the top three layers and they display a strong evolution over the remaining layers, giving them their typical ellipsoid shape.

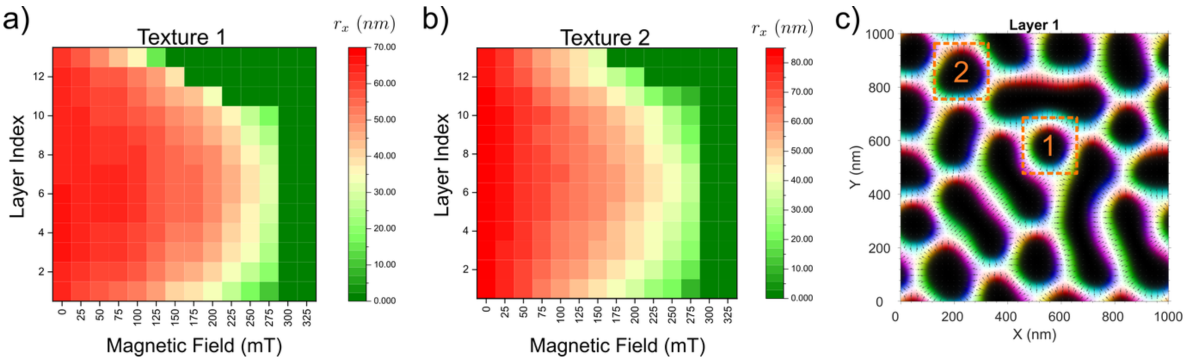

Figure S8: Typical evolution of the radius in SG structure depending on the field and the layer index for two different textures. For a given texture, the radius is taken as the distance from the centre to a cell in which $m_z = 0$. The textures under consideration are identified (1) (panel (a)) and (2) (panel (b)) on the magnetization cut on the right, corresponding to the bottom layer at 0 mT (panel (c)).

2D topological properties To study the topology of the skyrmionic cocoons, a 2D topological number W in each magnetic layer is computed using a lattice-based approach [2] at different fields for both the SG and the DG (Fig. S9a ,b). In the continuous approach, W is defined as:

$$W = \frac{1}{4\pi} \int \boldsymbol{m} \cdot (\partial_x \boldsymbol{m} \times \partial_y \boldsymbol{m}) \, \mathrm{d}\boldsymbol{r} \tag{2}$$

We start at a high OOP positive field and sweep towards OOP negative fields after relaxation every 25 mT, mimicking half of a hysteresis cycle. The conclusions presented hereafter are valid for the SG and the gradient parts of the DG. From positive field down to -100 mT, W remains constant at a negative value in the bottom layers up to the layer 6 (starting from the bottom) where it becomes close to zero then decreases back down in the last three layers. It reaches values greater than ± 1 because we have multiple objects in our simulation space and each of them contributes typically by ± 1 so the extremal value reflects the density. In the layers in which the dipolar and DMI fields nearly cancel each other [3], W drops to zero with domain walls uniformly pointing in the same direction (see red dotted square in layer 8 in Fig. S9c). The strong PMA layers corresponds to $W = -1$ as a single object with Néel walls is present in the simulation space. This simple 2D topological number already hints at interesting non-trivial properties for such complex objects.

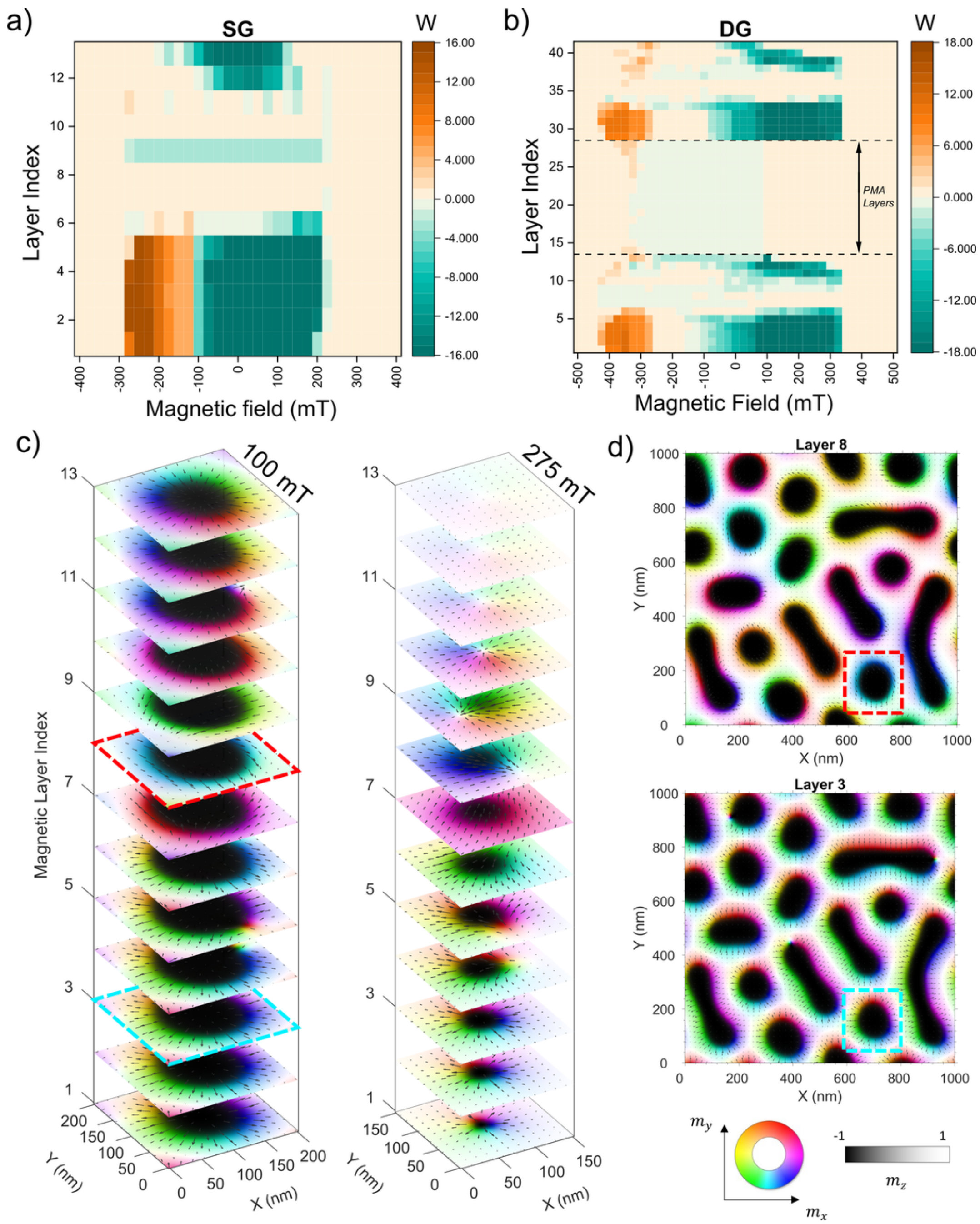

Figure S9: Numerical study of the topology. Lattice-based computation of the topological number for a a) SG and b) DG with a decreasing magnetic field. c) Magnetization cut of a typical texture in the different layers for a SG at 100 mT and 275 mT evidencing the field evolution of the texture from a skyrmion tube to a cocoon. d) Horizontal cut of the whole simulation space for the layers 3 and 8 at 100 mT. The square contours correspond to the one indicated in c). The color scale used in the various display for the magnetization is shown in d).

3D topological properties To further probe the topology of skyrmionic cocoons, we can consider the 3D generalization of the winding number [4, 5]:

$$q = \frac{1}{4\pi} \oiint_S \epsilon_{ijk} \boldsymbol{m} \cdot \partial_j \boldsymbol{m} \times \partial_k \boldsymbol{m} \, \mathrm{d}A_i \tag{3}$$

Where the integral is taken over a surface S, enclosing the structure. For a single enclosed Bloch point, q takes the value ± 1, and q can be zero for even number of Bloch points, like for the dipole strings, also called torons or globules. In our case, when the cocoon is fully buried into the multilayer, the magnetization on a contour is quasi uniform without no curvature, and therefore leads to $q = 0$ (see Fig. S10a). Alternatively, if the cocoon 'emerges' at the surface of the multilayer, with a skyrmion in the topmost (or bottommost) magnetic layer, then q is entirely determined by the 2D skyrmion number of this last layer (Fig. S10b) and is thus non-zero. However, we stress that the lack of continuity of the magnetization, especially along the vertical direction, breaks the premises of the underlying mathematical foundations: in particular, $q \neq 0$ is not associated with the presence of Bloch points, which are 'hidden' between the magnetic layers. As illustrated in Fig. S10c, when the symmetric exchange is low enough and the layers only coupling through dipolar exchange, there is no more continuity of the magnetization texture along the out-of-plane direction. Therefore, the name 'skyrmionic cocoons' is introduced to indicate this fundamental difference with the continuous textures called torons.

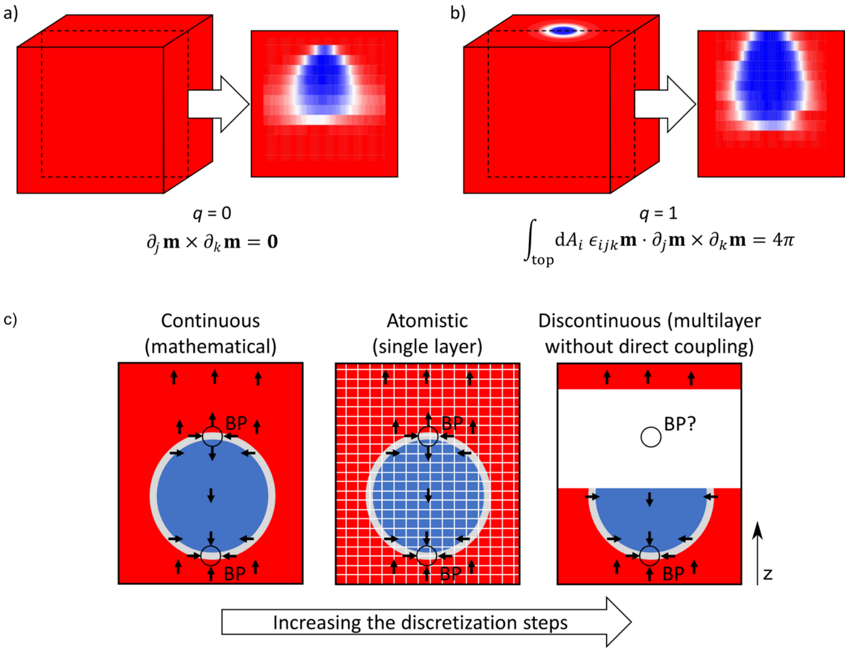

Figure S10: Calculation of the 3D topological charge, neglecting the non-continuity of the magnetization along the growth direction. a) Selection of a cubic bounding box around a cocoon fully buried in the magnetic multilayer. A slice of the magnetization inside the box, indicated by the dashed line, is shown next to the bounding box. b) When the cocoon emerges to the surface of the magnetic multilayer, one side of the box displays a skyrmion texture, yielding a topological charge of ± 1. c) What discretization preserves the 3D topology? As the symmetric exchanged is reduced, keeping only the dipolar coupling between the layers, no more continuity of the magnetization is expected between layers, and the Bloch points can be truly avoided.

Supplementary References

[1] Arne Vansteenkiste, Jonathan Leliaert, Mykola Dvornik, Mathias Helsen, Felipe Garcia-Sanchez, and Bartel Van Waeyenberge. The design and verification of mumax3. *AIP advances*, 4(10):107133, 2014.

[2] Joo-Von Kim and Jeroen Mulkers. On quantifying the topological charge in micromagnetics using a lattice-based approach. *IOP SciNotes*, 1(2):025211, 2020.

[3] William Legrand, Jean-Yves Chauleau, Davide Maccariello, Nicolas Reyren, Sophie Collin, Karim Bouzehouane, Nicolas Jaouen, Vincent Cros, and Albert Fert. Hybrid chiral domain walls and skyrmions in magnetic multilayers. *Science advances*, 4(7):eaat0415, 2018.

[4] Mi-Young Im, Hee-Sung Han, Min-Seung Jung, Young-Sang Yu, Sooseok Lee, Seongsoo Yoon, Weilun Chao, Peter Fischer, Jung-Il Hong, and Ki-Suk Lee. Dynamics of the bloch point in an asymmetric permalloy disk. *Nature communications*, 10(1):1–8, 2019.

[5] Oleksandr V Pylypovskyi, Denis D Sheka, and Yuri Gaididei. Bloch point structure in a magnetic nanosphere. *Physical Review B*, 85(22):224401, 2012.
